# Stress among university students: factorial structure and measurement invariance of the Italian version of the Effort-Reward Imbalance student questionnaire

**DOI:** 10.1186/s40359-019-0343-7

**Published:** 2019-10-26

**Authors:** Igor Portoghese, Maura Galletta, Fabio Porru, Alex Burdorf, Salvatore Sardo, Ernesto D’Aloja, Gabriele Finco, Marcello Campagna

**Affiliations:** 1 Dipartimento di Scienze Mediche e Sanità Pubblica, Università degli Studi di Cagliari, SS554 bivio per Sestu, 09042 Monserrato, CA Italy; 2 Department of Public Health, Erasmus University Medical Center, Rotterdam, Netherlands

**Keywords:** Student stress, ERI, Effort, Reward, Overcommitment, Factorial validity, Invariance

## Abstract

**Background:**

In the last decade academic stress and its mental health implications amongst university students has become a global topic. The use of valid and theoretically-grounded measures of academic stress in university settings is crucial. The aim of this study was to examine the factorial structure, reliability and measurement invariance of the short student version of the effort-reward imbalance questionnaire (ERI-SQ).

**Methods:**

A total of 6448 Italian university students participated in an online cross-sectional survey. The factorial structure was investigated using exploratory factor analysis and confirmatory factor analysis. Finally, the measurement invariance of the ERI-SQ was investigated.

**Results:**

Results from explorative and confirmatory factor analyses showed acceptable fits for the Italian version of the ERI-SQ. A modified version of 12 items showed the best fit to the data confirming the 3-factor model. Moreover, multigroup analyses showed metric invariance across gender and university course (health vs other courses).

**Conclusions:**

In sum, our results suggest that the ERI-SQ is a valid, reliable and robust instrument for the measurement of stress among Italian university students.

## Background

In the last decade, there has been a growing attention in investigating stress risk factors and well-being consequences among university student’s population [1, 2]. Stress and mental health of university students is a crucial public health subject as healthy students will be the healthier workers of the future. Attending university has the potential to become a positive and satisfying experience for students’ life. However, there is empirical evidence that being a student may become a stressful experience [1, 3–6]. Stallman and Hurst [2] distinguished between eustress, important for student motivation and success at university, and distress, harmful for student’s well-being, as it exposes to a higher risk of psychological (for example, anxiety and burnout), behavioral (for example eating disorders), physical health problems (for example, ulcers, high blood pressure, and headaches), and suicidal ideation [7–10]. Furthermore, many scholars found that high stress was linked to reduced academic performance, low grade averages, and low rates of graduation and higher dropout [11–15].

Academic stressors have been identified as including high workload, attending lessons, respecting deadlines, balancing university and private life, and economic issues. Those stressors are linked to a greater risk of distress and reduced academic achievement [1, 16–19].

Many authors adopted and extended original measures of stress, for example, by adapting work related stress measures to the university context [20, 21]. Most of these measures were designed for medical students [22] or employed measures of stress not specifically developed for the academic context [20–22].

According to Hilger-Kolb, Diehl, Herr, and Loerbroks [23], the vast majority of these measures lack a stress theoretical model. It may represent an important limitation as, meausers based on a common tested stress model may be better help researchers to capture the links between stress and health among university students and to develop theory-based interventions [21]. Effort-Reward Imbalance (ERI) [24] is among the most common tested and valid models of stress. According to this model, when high efforts are balanced by low rewards, the resulting imbalance may generate negative emotions and sustained stress experiences. Originally developed to investigate stress risks among workers, this model has been the theorethical root of many studies investigating stress in non-working contexts.

Recently, Wege, Muth, Angerer, and Siegrist [25] extended the original ERI model to the context of university and adapted the ERI short questionnaire to the university setting, showing good psychometric properties. Thus, according to this theoretical approach, students’ stress was defined as the result of an imbalance between effort, such as high study load, and reward, such as being respected from supervisors.

A vast number of empirical studies measuring effort–reward imbalance in workplace context confirmed good psychometric qualities of the ERI short questionnaire [26, 27]. Furthermore, psychometrically validated versions have been tested in 9 languages and in large European cohort studies, confirming the good psychometric qualities of the short ERI [28, 29].

Concerning the student version of the ERI, there is limited psychometric information available. Given the importance of academic stress for understanding students’ mental health risk, the aim of this study was to investigate the psychometric properties of the Italian version of the ERI-student questionnaire [25]. To address this goal, we examined the factor structure of the Italian version of the ERI-SQ, assessed internal consistency for the dimensions of effort, reward, and over-commitment, and test the measurement invariance of the ERI-SQ.

## Methods

### Participants and procedure

The study population (convenience sample) was recruited through a public announcement at electronic learning platforms for students and university students’ associations’ network that contained an invitation for participating in a “Health Promoting University” survey. The online survey was implemented with Limesurvey from October 16th, 2017 to November 27th, 2017 and was restricted to enrolled university students (bachelor level and master level). The survey’s homepage reported the online informed consent form with specific information about study purpose, general description of the questionnaire, including information about risks and benefits of participation. Also, the time necessary to complete the survey (less than 10 min) and privacy policy information were reported. Specifically, to ensure anonimity, we did not register ip address neither requested any another sensitive data. The investigators and research team did not employ any active advertising to increase recruitment rates neither played any active role in selecting and/or targeting specific subpopulations of respondents. A total of 9883 students agreed to participate in the survey with 6448 (65.24%) completing the survey (target population: 1.654.680 Italian university students in 2017). The Italian version of the ERI-SQ (see Table 4 in Appendix) was translated following the back-translation procedure [30].

### Demographics

The sample for this research consisted of 75.5% females (*n* = 4869). Participants in this study ranged from 19 to 56 years of age, M = 22.97, SD = 3.01. 56.2% (3624) were enrolled in bachelor prrogrammes and 43.8% (2824) in master programmes. 39.6% (2551) were enrolled in health related courses (such as medicine, nursing, psychology, and biomedical science).

### Measures

Stress was assessed with the ERI-SQ [25] that was developed for use in student samples. The version adopted in this study consists of 14 items that constitute three scales: Effort (EFF; 3 items; example: “I have constant time pressure due to a heavy study load”), Rewards (REW; 6 items; example: “I receive the respect I deserve from my supervisors/teachers”), and over-commitment (OC; 6 items; example: “As soon as I get up in the morning I start thinking about study problems”). All items are scored on a 4-point rating scale ranging from 1 (strongly disagree) to 4 (strongly agree). Average scores of items ratings for each subscale were calculated following appropriate recoding.

### Statistical analyses

Statistical analyses were performed with R [31] and Rstudio [32]. The factorial structure was investigated using exploratory factor analysis (EFA; psych package) [33] and confirmatory factor analysis (CFA; lavaan package) [34]. The dataset was randomly split in half to allow for independent EFA (training set) and CFA (test set). A robust ML estimator was used for correcting violations of multivariate normality.

The analyses were conducted in two stages. Firstly, an EFA with principal axis factor (PAF) analysis was performed. Using Horn’s Parallel Analysis for factor retention. Internal consistency was assessed via Cronbach’s alpha coefficient.

The second stage of analysis involved investigating the factor structure of the Italian version of the ERI-SQ, a series of CFA were performed. As Mardia’s test of multivariate kurtosis (28.78, *p* < .0001) showed multivariate non-normality, we investigated model fit with robust maximum likelihood (MLM) [35]. We compared alternative models: a 1-factor model, in which all 14 items were assessed as one common factor, a 3-factor model where items reflected the three subscales of the ERI-SQ, and a three-factor model with adjustments made according to error theory. We considered several fit indices: χ2(S-B χ2) [36], the robust root mean square error of approximation (RMSEA); the standardized root mean square residual (SRMR) and the robust comparative fit index (CFI). For CFI, score > .90 indicated acceptable model fit. For both RMSEA and SRMR, score ≤ .05 was considered a good fit, and ≥ .08 a fair fit [37, 38].

Finally, the measurement invariance of the ERI-SQ was investigated. We performed a series of multi-group CFAs. We tested 5 nested models with progressive constrained parameters: Model 0 tested for configural invariance; Model 1 tested for metric invariance (constrained factor loadings); Model 2 tested for scalar invariance (constrained factor loadings and item intercepts); Model 3 tested for uniqueness invariance (constrained factor loadings, item intercepts, and residual item variances/covariances); Model 4 tested for structural invariance (constrained factor loadings, item intercepts, and factor variances/covariances). Models were compared by using the chi-square (χ2) [39]. In comparing nested models, we considered changes in CFI, RMSEA, and SRMR indices as follows: ΔCFI ≤ − 0.02 [40, 41], ΔRMSEA ≤0.015, and ΔSRMR ≤0.03 for tests of factor loading invariance [40, 42] and ΔCFI ≤-0.01, RMSEA ≤0.015, and SRMR ≤0.01 for test of scalar invariance [42].

## Results

### Exploratory factor analysis

We split the dataset (*n* = 6448) into random training and test samples. EFA was performed on the training sample (*n* = 3879). Results from parallel analysis with 5000 parallel data sets using 95th percentile random eigenvalue showed that the eigenvalues for the first three factors exceeded those generated by the random data sets. Subsequently, a three-factor solution was inspected in a principal axis factor analysis with varimax rotation on the 14 items of the ERI-SQ (Table 1).

**Table 1:** Factor patter matrix for the Italian version of the ERI-SQ

	Effort		Reward		Overcommitment	
	EFA	CFA	EFA	CFA	EFA	CFA
EFF1	0,73	0,80^*^				
EFF3	0,49	0,56^*^				
REW1			0,71	0,62^*^		
REW3r			0,57	0,56^*^		
REW5			0,52	0,61^*^		
REW2			0,41	0,41^*^		
REW6			0,34	0,36^*^		
OC4					0,83	0,82^*^
OC1					0,60	0,73^*^
OC5					0,59	0,58^*^
OC2r					0,54	0.61^*^
OC3					0,42	0,51^*^

*EFA* Explorative Factor Analysis; *n* = 3224. Loading below ǀ.30ǀ have been suppressed

*CFA* Confirmative Factor Analysis; *n* = 3224; ^*^ *p* < .01

The EFA revealed that two items (EFF2 “I have many interruptions and disturbances while preparing for my exams” and REW4r “ I am not sure whether I can successfully accomplish my university trainings”) loaded on the same factor. An item analysis revealed that, probably, both items have a general and ambiguous formulation among student population. These items were therefore deleted from all analyses, as subsequent analyses were conducted with the remaining 12 items. We then re-conducted a principle axis factor analysis with varimax rotation. The three factors collectively explained 40.0% of the variance in the three facets. After rotation, the factors were interpreted as effort, reward and over-commitment.

### Confirmatory factor analysis

Based on the results from the EFA, three models were tested on the test sample (*n* = 3879; Table 2).

**Table 2:** Fit Indices of the MBI-GS Students from the CFA

Model	S-Bχ2	df	ΔS-Bχ2	Δdf	p	rCFI	rTLI	rRMSEA	SRMR
One-factor model	1833.95	54				.78	.73	.109	.084
Three-factor model	384.17	51	1449,79	3		.96	.95	.048	.033

*n* = 3224; *S-Bχ2* Satorra-Bentler scaled chi-square, *rCFI* robust Comparative Fit Index, *rTLI* robust Tucker Lewis Index, *RMSEA* Robust Root Mean Square Error of Approximation, *SRMR* Standardized Root Mean Residual

Fit indices for the unidimensional model S-Bχ2(54) = 1833.95, rCFI = .78, rTLI = .73, RMSEA = .109, SRMR = .084 suggested that the model did not provide a good fit to the data. We next considered the three-factor model [21]. Fit indices suggested this model fits the data well, S-Bχ2(51) = 384.17, rCFI = .96, rTLI = .95, rRMSEA = .048, SRMR = .033. The χ2 difference test was significant, ΔS-Bχ2(3) = 1449.79, *p* < .001. All standardized factor loadings were significant.

Internal consistency was .66 for reward, and .78 for overcommitment. Correlations between the three latent factors were as follows: −.30 between effort and reward, .52 between effort and over-commitment, −.33 between reward and over-commitment. Mean scores were: effort = 3.04 (SD = 0.59), reward = 2.67 (SD = 0.48) and over-commitment = 2.65 (SD = 0.63). The mean value of the effort-reward ratio was 1.20 (SD = 0.41).

### Measurement invariance

Next, for testing measurement invariance, we conducted a series of multi-group CFAs across different groups: health (medicine, nursing, etc.) vs other courses (engineering, economy, etc.) and gender (male vs female).

First, a series of multi-group CFA (MGCFA) was conducted on the health and other university courses. Table 3 shows that configural invariance was supported (Model 0) as fit the data well across health courses (*n* = 2551) and other courses (*n* = 3897): S-Bχ2(102) = 398.06, CFI = .962, RMSEA = .045, SRMR = .032. All loadings were significant (*p* < .01). We found support for metric invariance (Model 1): ΔCFI = −.001, ΔRMSEA = −.001, and ΔSRMR = −.002. Next, we did not find support for scalar invariance (Model 2; ΔCFI = − .043; ΔRMSEA = .019, and ΔSRMR = .017). As full scalar invariance was not supported, we tested for partial invariance. Inspecting modification indices, we found that three items from the reward subscale (REW2 “I receive the respect I deserve from my fellow students”; REW3 “I am treated unfairly at university”; and REW6 “Considering all my efforts and achievements, my job promotion prospects are adequate”) and all items from the over-commitment subscale lacked invariance. However, as showed on Table 3, partial scalar invariance (Model 2b) was not supported (ΔCF = −.021, ΔRMSEA = −.012, and ΔSRMR = .011).

**Table 3:** Test of invariance of the proposed three-factor structure of the ERI-SQ between health courses (*n* = 2551) and other courses (*n* = 3897) students, and female (*n* = 4869) vs male students (*n* = 1579): results of multigroup confirmatory factor analyses

Model	S-Bχ2	df	rCFI	rRMSEA	rSRMR	Nested Model	ΔrCFI	ΔrRMSEA	ΔrSRMR
Health vs other courses									
Health	178.44	51	.959	.046	.032				
Non-Health	218.51	51	.963	.041	.032				
M0. Configural invariance	398.06	102	.962	.045	.032				
M1. Metric invariance	417.12	111	.961	.044	.035	M1-M0	−.001	−.001	.002
M2. Scalar invariance	822.39	120	.912	.063	.052	M2-M1	−.043	.019	.017
Female vs male students									
Female students	303.65	51	.956	.045	.032				
Male students	141.59	51	.955	.047	.036				
M0. Configural invariance	445.20	102	.956	.049	.033				
M1. Metric invariance	465.98	111	.955	.047	.036	M1-M0	−.001	−.002	.003
M2. Scalar invariance	547.82	120	.946	.050	.038	M2-M1	−.009	.003	.002
M3. Uniqueness invariance	576.19	132	.941	.049	.040	M3-M2	−.005	−.001	.002
M4. Structural invariance	666.14	135	.931	.053	.052	M4-M3	−.010	.004	.012

*df* degrees of freedom, *CFI* Comparative Fit Index, *RMSEA* Root Mean Square Error Of Approximation, *SRMR* Standardized Root Mean Square Residual

Next, we performed a series of MGCFAs to test the invariance of the ERI-SQ between female and male students (Table 3). We found support for configural invariance (Model 0) across female (*n* = 4869) and male (*n* = 1579) groups: S-Bχ2(102) = 445.20, CFI = .956, RMSEA = .049, SRMR = .033. All loadings were significant (*p* < .01). Next, we found support for metric invariance (Model 1): ΔCFI = − .001, ΔRMSEA = −.002, and ΔSRMR = .003. Next we found support for scalar invariance (Model 2): ΔCFI = −.009, ΔRMSEA = .003, and ΔSRMR = .002. Next uniqueness invariance (Model 3) was supported: ΔCFI = −.005, ΔRMSEA = −.001, and ΔSRMR = .002. Finally, we found support for structural invariance (Model 4): ΔCFI = −.010, ΔRMSEA = .004, and ΔSRMR = .012.

## Discussion

The main objective of this study was to examine the factorial validity and invariance of the Italian version of the ERI-SQ among Italian university students. Overall, our results confirmed the factorial structure underlying the ERI-SQ, as theorized by Siegrist [25] and reported by Wege and colleagues [25] in the student version of the ERI. However, in light of the conclusions drawn from the EFA, to enhance the fit of the model, we had to delete two items with high cross loadings. The deleted items were problematic in the Wege and colleagues [25] study too. Specifically, both items (EFF2 and REW4) showed a low factor loading in the CFA.

In the Italian sample, using a modified and shortened version (12 items) of the ERI-SQ, we confirmed the three factors structure components of the model, showing a satisfactory fit of the data structure with the theoretical concept. In sum, the current findings show that the ERI-SQ is as a reliable instrument for measuring academic stress among students.

Finally, as expected, we found support for metric invariance across gender and university course, health (medicine, nursing, etc.) vs other courses (engineering, economy, etc.). Mainly, MCFAs confirmed that the three-factor structure of the ERI-QS is (mostly) invariant across different groups. More specifically, we found support for parameter equivalence across gender (structural invariance), but the ERI-SQ was significantly different in health vs other courses. In fact, we were not able to find scalar invariance, suggesting that items REW2, REW3, REW6 and all the over-commitment items vary by academic courses. However, the lack of scalar invariance is a negligible issue for the Italian version of the ERI-SQ.

### Implications and limitations

Results from our study showed that the Italian version of the ERI-SQ-10 provides a psychometrically sound measure of stress as defined in the ERI theoretical framework. The ERI-SQ is a brief and easy to administer university student stress measure. In this sense, using valid and reliable measures of stress is crucial for Italian university counselling services to advance in monitoring and understanding the levels of stress affecting students and how to support them. In this manner it would be possible to offer appropriate mental health support [43] when students are exposed to lack of reciprocity between spending high efforts and receiving low rewards during their student career.

The present study has several limitations. First, data were obtained from a convenience sample offering reduced generalizability of our results. However, for the purpose of the study this sample was deemed appropriate. Second, the Effort dimension was composed of only two items. A factor with only two items leads to a CFA that cannot be estimated unless constraining the model. Future research would overcome this limitation by reevaluating a wider version of the ERI and adapting other items from the Effort factor as defined in the ERI questionnaire [24]. Third, further research is also recommended concerning construct and criterion validity [44]. Specifically, we are not able to provide evidence of convergent validity (how closely the ERI-SQ is related to other variables and other measures of the same construct), and discriminant (ERI-SQ does not correlate with other variables that are theoretically not related). Future research would consider to analyse it by employing a multitrait-multimethod [45]. Finally, as one of the anonymous reviewers correctly pointed out, our study does not offer any evidence of criterion validity, mainly concurrent validity (the degree to which a measure correlates concurrently to an external criterion in the same domain [44]. However, according to Wege and colleagues [25], no studies have provided estimates of these validities for the ERI-SQ. Future research would provide evidence of it by analyzing the correlation between the ERI-SQ and a theoretically similar measure of student stress. In this sense, concurrent validity is an important area of future research. Fourth, we did not test for test–retest reliability. Future research should address these issues. Despite these important limitations, the Italian version of the ERI-SQ showed satisfactory psychometric properties.

## Conclusions

In the present study, we found that the Italian version of the ERI-QS partially confirms the original version from Wege and colleagues [25]. We were able to show satisfactory psychometric properties of the ERI-SQ. Considering a high prevalence of academic distress among University students and the limited interventions aimed to reduce stress [46], universities should employ preventive interventions by measuring and controlling for potentially harmful psychosocial risk. In this sense, the Italian version of the ERI-QS presents a valid instrument for measuring academic stress on Italian-speaking university students.

## Data Availability

Raw data pertaining to analyses performed in this study are available available from the authors upon reasonable request.

## Appendix

**Table 4:** Italian version of the ERI-SQ

EFF1	Sono costantemente sotto pressione a causa dell’eccessivo carico di studio.
EFF3	Il mio studio è diventato sempre più impegnativo.
REW1	Sono trattato dai miei docenti con il rispetto che merito.
REW3r	Sono trattato in modo ingiusto all’università.
REW5	Considerando tutti i miei forzi, ricevo l’apprezzamento che merito.
REW2	Sono trattato dai miei colleghi con il rispetto che merito.
REW6	Considerando i miei sforzi ed i risultati raggiunti, le mie prospettive di lavoro sono adeguate.
OC4	Raramente riesco a non pensare allo studio; è ancora nella mia mente quando vado a dormire
OC1	Appena mi alzo al mattino comincio a pensare ai problemi legati allo studio
OC5	Se rimando qualcosa che avrei dovuto fare nella giornata, non riesco più a dormire per la preoccupazione
OC2r	Quando torno a casa, mi rilasso facilmente e “stacco” dallo studio
OC3	Le persone a me vicine dicono che mi sacrifico troppo per lo studio

Answer format—4-point Likert scale: [1] ‘strongly disagree’, [2] ‘disagree’, [3] ‘agree’, [4] ‘strongly agree’

r Reversed items: [1] ‘strongly agree’, [2] ‘agree’, [3] ‘disagree’, [4] ‘strongly disagree’

## Abbreviations

CFA: Confirmatory Factor Analysis
CFI: Comparative Fit Index
EFA: Exploratory Factor Analysis
EFF: Effort
ERI: Effort-Reward Imbalance
ERI-SQ: Effort-Reward Imbalance Students Questionnaire
MGCFA: Multi-Group Confirmatory Factor Analysis
ML: Maximum Likelihood
MLM: Robust Maximum Likelihood
OC: Over-commitment
PAF: Principal Axis Factor
REW: Rewards
RMSEA: Root Mean Square Error of Approximation
SD: Standard Deviation
SRMR: Standardized Root Mean Square Residual
